# Supplementary figures and images for: Design, development, and evaluation of gene therapeutics specific to KSHV-associated diseases
Source: bioRxiv. 2025 Feb 19:2025.02.19.639178. Preprint. [Version 1] doi: 10.1101/2025.02.19.639178 (PMC11870588; doi:10.1101/2025.02.19.639178)

**a**

pAAV-TR2-OriP-mCardinal vector

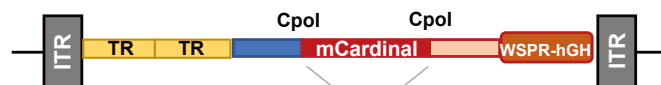

pAAV-TR2-OriP-TK vector

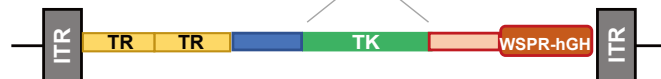**b**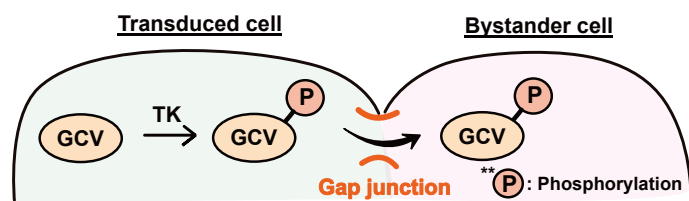

**a**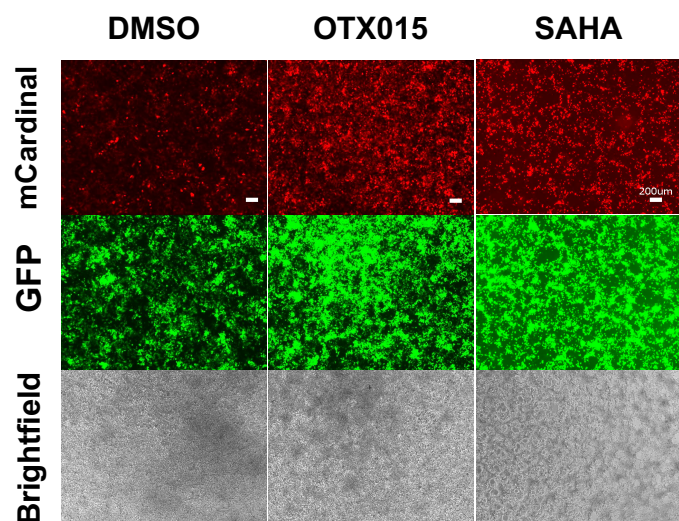**b**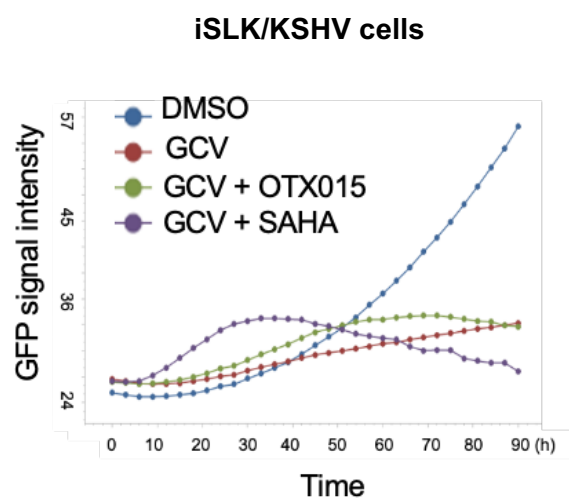

Supplement: Supplement 1 — Supplementary Figure 1. Construction of pAAV-TR2-OriP-TK vector. a. Schematic diagram of pAAV-TR2-OriP-TK vector. mCardinal sequence encoding in the pAAV-TR2-OriP-mCardinal vector was replaced with TK sequence. TR: terminal repeat, TK: thymidine kinase, ITR: inverted terminal repeat. b. Schematic diagram of TK/GCV system. TK phosphorylates the prodrug ganciclovir (GCV) into a toxic nucleotide analog, leading to selective cell death in TK-expressing cells. The phosphorylated GCV can also diffuse into neighboring bystander cells by gap junction, inducing cytotoxic effects even in non-TK-expressing cells Supplementary Figure 2. SAHA and OTX015 stimulate transcription from the TR2-OriP vector. a. Fluorescent and bright field cell images. KSHV-infected 293 cells were seeded in 12 well plates and transduced with AAV8-TR2-OriP-mCardinal. Two days after AAV8-TR2-OriP-mCardinal infection, cells were treated with mock (DMSO), OTX015 (200 nM), or SAHA (1 μM). Images were taken four days after the AAV infection. Scales: 200 μm. b. KSHV-infected 293 cell growth. KSHV-infected 293 cells were seeded in 6 well plates, and GCV (10 μg/ml) with or without OTX015 (200 nM) or SAHA (1 μM) were added to cells two days after AAV8-TR2-OriP-TK infection. Cell growth (upper) and GFP signal intensity (lower) were continuously monitored by Incucyte for 90 hours. [file media-1.pdf]
